# Utilization of Nanoparticles for Treating Age-Related Macular Degeneration

**DOI:** 10.3390/ph18020162

**Published:** 2025-01-25

**Authors:** Anna Nikolaidou, Ellas Spyratou, Athanasia Sandali, Theodora Gianni, Kalliopi Platoni, Lampros Lamprogiannis, Efstathios P. Efstathopoulos

**Affiliations:** 1 Medical School, National and Kapodistrian University of Athens, 11527 Athens, Greece; 2 2nd Department of Radiology, Medical School, National and Kapodistrian University of Athens, 11527 Athens, Greecestathise@med.uoa.gr (E.P.E.); 3 Medical School, Aristotle University of Thessaloniki, 54124 Thessaloniki, Greece; 4 Ophthalmica Microsurgery Institute, 54655 Thessaloniki, Greece

**Keywords:** nanoparticles, nanotechnology, retinal degeneration, blindness, ocular drug delivery, ophthalmology, age-related macular degeneration

## Abstract

Age-related macular degeneration (AMD) is a predominant cause of vision loss, posing significant challenges in its management despite advancements such as anti-vascular endothelial growth factor (anti-VEGF) therapy. Nanomedicine, with its novel properties and capabilities, offers promising potential to transform the treatment paradigm for AMD. This review reports the significant advancements in the use of diverse nanoparticles (NPs) for AMD in vitro, in vivo, and ex vivo, including liposomes, lipid nanoparticles, nanoceria, nanofibers, magnetic nanoparticles, quantum dots, dendrimers, and polymer nanoparticles delivered in forms such as gels, eye drops, intravitreally, or intravenously. Drug delivery was the most common use of NPs for AMD, followed by photodynamic therapy dose enhancement, antioxidant function for nanoceria, biomimetic activity, and immune modulation. Innovative approaches arising included nanotechnology-based photodynamic therapy and light-responsive nanoparticles for controlled drug release, as well as gene therapy transfer. Nanomedicine offers a transformative approach to the treatment and management of AMD, with diverse applications. The integration of nanotechnology in AMD management not only provides innovative solutions to overcome current therapeutic limitations but also shows potential in enhancing outcomes and patient quality of life.

## 1. Introduction

At least 2.2 billion individuals worldwide suffer from vision impairment according to the World Health Organization, including 8 million with age-related macular degeneration [1], with these numbers expecting to rise without additional investment in global eye health. This high global prevalence, along with an increased prevalence of eye diseases in the aging population, underscores the importance of developing innovative therapies that target disease at the cellular and molecular levels to prevent or slow the progression of vision loss. Such targeted delivery can be achieved with nanoparticles used as delivery agents and allows for a sustained therapeutic effect with minimal systemic exposure, reducing adverse effects and enhancing drug bioavailability within ocular tissues. This targeted approach is particularly valuable in addressing retinal diseases like AMD, one of the most prevalent causes of visual impairment [2], where precise delivery to affected tissues could significantly slow or prevent disease progression. In this sense, advancing early detection and effective management of these pathological conditions, which, if left untreated or undiagnosed, can lead to irreversible vision loss, is crucial.

AMD is a degenerative disease that affects individuals older than 55 years and primarily affects the macula, the central area of the retina, which is responsible for clear vision and the ability to distinguish fine details, making it difficult to read, drive, and recognize faces [3]. The effects of AMD in the macula lead to central vision loss and a subsequent reduction in sharpness and clarity in the central visual field. Meanwhile, the peripheral vision remains largely intact. AMD is the leading cause of irreversible vision loss among older adults [4]. This disease represents a significant cause of central vision loss among individuals aged 50 and above. The prevalence of this condition is 200 million worldwide and increases with age [2]. Thus, AMD is more prevalent in developed regions, where life expectancy is higher. Furthermore, there is an increased risk associated with female gender, family history, and race [5]. Studies have shown that Caucasians are more likely to develop any form of AMD compared to those of Asian and African descent. Additionally, Caucasians demonstrated a heightened likelihood of developing geographic atrophy in comparison to Asians, Hispanics, and Africans [2].

AMD constitutes a multifactorial retinal disorder influenced by aging, smoking, genetic predisposition, nutritional deficiencies, hypertension, and hyperlipidemia, which together contribute to macular degeneration [6]. AMD is classified into non-neovascular (dry) and neovascular (wet) forms, with dry AMD characterized by gradual drusen accumulation and slower vision loss, while wet AMD involves abnormal blood vessel growth, leading to rapid and severe damage. Pathophysiology includes retinal pigment epithelium (RPE) dysfunction, oxidative stress, drusen-induced inflammation, and vascular endothelial growth factor (VEGF)-driven angiogenesis [7,8,9,10,11]. Diagnosis relies on visual acuity tests, fundoscopy, OCT, and sometimes angiography. While non-neovascular AMD lacks a cure, its progression can be delayed with nutritional supplements and lifestyle changes [12,13]. Neovascular AMD treatment focuses on anti-VEGF injections and occasionally photodynamic therapy to inhibit abnormal blood vessel growth, aiming to stabilize and improve vision [14,15,16,17,18]. However, traditional treatments with regular intravitreal injections for AMD provide limited improvement. Nanomedicine offers promising potential to transform the treatment paradigm for AMD [19]. Nanoparticle-based drug delivery systems enhance treatment efficiency. Prospects include precision therapy and reducing side effects. Nanoparticles are being employed for improved ocular drug delivery. Their ability to be designed to overcome the eye’s physical and anatomical barriers makes them highly effective for ocular applications. Notably, the eye is considered an immune-privileged site, meaning it has a unique immune environment that limits the intensity of immune responses to avoid damage to delicate ocular tissues [20]. This quality allows engineered nanoparticles to interact with ocular structures more effectively without eliciting a strong immune reaction, enhancing their potential for sustained and targeted therapeutic delivery. In this narrative review, the relevant literature of the current state of nanomedicine in the management of AMD is explored, assessing how different nanomaterials and delivery strategies are investigated for AMD treatment.

## 2. Nanoparticles for AMD Treatment

**Figure 1:**
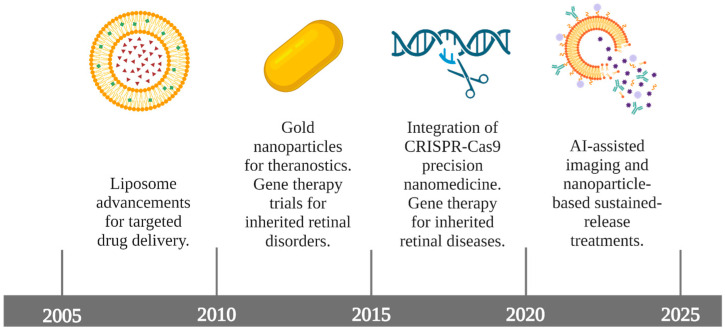
The literature on AMD and NPs through the years.

Many research groups have conducted in vitro experiments to determine the stability, permeability, cytotoxicity, and ocular tolerance of potential NPs to use in AMD treatment. Figure 1 describes the literature through major timepoints in the research around NPs and applications in AMD.

**Figure 2:**
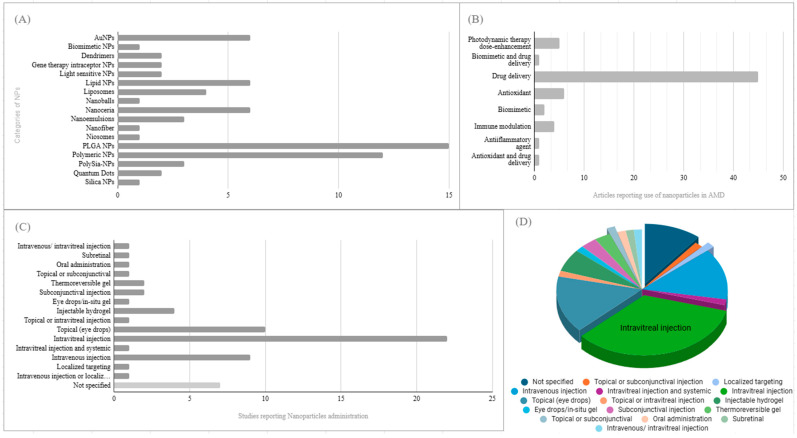
(**A**) Nanoparticles used in AMD treatment; (**B**) Functions of nanoparticles for AMD managements; (**C**) Ways of administration of nanoparticles in tabular form; (**D**) pie chart illustration of administration ways.

In vivo animal studies using mostly CNV models have also been performed to elucidate whether NPs can produce a therapeutic effect against wet AMD characterized by angiogenesis. Studies using combined design in vitro and/or in vivo and/or ex vivo designs are included in the in vivo section of this review. PLGA NPs, polymeric NPs, lipid NPs, and nanoceria were among the most reported NPs used. The extensive list of NPs used can be shown in Figure 2.

**Figure 3:**
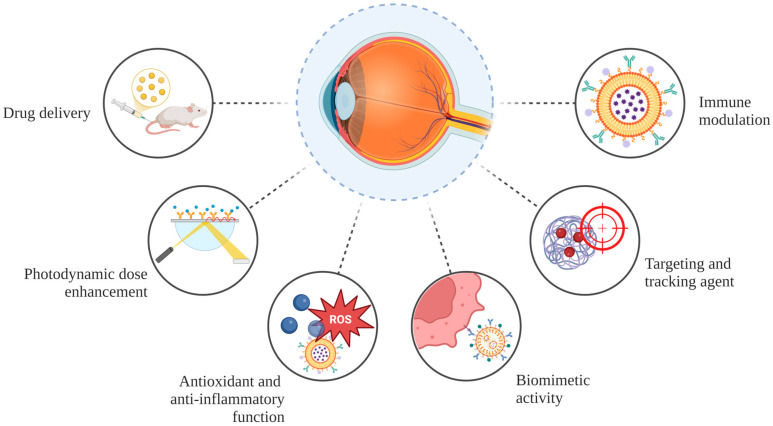
Nanoparticles’ use in AMD therapy.

Drug delivery was the most common use of NPs for AMD, followed by photodynamic therapy dose enhancement, antioxidant function for nanoceria, biomimetic activity, and immune modulation (Figure 2B). The use of NPs in the treatment of AMD is illustrated in Figure 3. As for the way of administration proposed in the studies, most reported intravitreal injections, while others suggested intravenous and topical administration with eye drops (Figure 2C).

### 2.1. In Vivo Studies

#### 2.1.1. Lipid Nanoparticles In Vivo

Lipid NPs are spherical, with a diameter between 10 and 1000 nm, and they contain lipid moieties. They possess a solid lipid core and a matrix of soluble lipophilic molecules, and their external core is stabilized by surfactants or emulsifiers. Canioni et al. (2021) [21] evaluated dexamethasone palmitate (DXP)-loaded nanoparticles (~35 nm) for intravitreal injection in a pigmented rabbit model of neovascular AMD. The lipid nanoparticles, stabilized with Pluronic F127, showed high encapsulation efficacy (99%) and effectively restored the blood–retinal barrier (BRB) for up to 1 month, although the effect declined by 2 months due to rapid clearance of the small-sized particles. No significant adverse effects were reported, and the study highlighted the potential of DXP NPs as a stable formulation with minimal visual disturbance for AMD treatment. Lipid-based nanodispersion was developed by Ponnusamy et al. (2019) [22] (12–26 nm, spherical) and was co-loaded with artemisinin and dexamethasone for the topical treatment of wet AMD. The nanoparticles (12–26 nm, spherical, zeta potential −6 to −10 mV) demonstrated controlled drug release and effective anti-angiogenic properties, with an up to 103% release of dexamethasone in vitro. The formulation showed good ocular bioavailability, acceptable safety levels, and promising results in both in vitro and chick embryo (CAM) models, highlighting its potential for improved corneal permeability and AMD management. Mei et al. (2022) [23] introduced synthetic high-density lipoprotein (sHDL) nanoparticles, made from ApoA-I mimetic peptide and DMPC phospholipids, to deliver rapamycin intravitreally, achieving a 125-fold increase in solubility, reduced NF-κB-mediated inflammation, and enhanced targeting of the RPE layer in rats. The sHDL nanoparticles demonstrated stability, rapid retinal diffusion, and no toxicity within 72 h of injection. The study demonstrated their rapid retinal diffusion and non-toxicity at doses of 10–100 μg/mL in ARPE-19 cells, confirmed through in vitro studies on ARPE-19 cells and in vivo experiments in Sprague Dawley rats.

Only a few studies investigated NPs for dry AMD, among which Sun et al. (2020) [24] developed astragaloside-IV lipid nanocapsules (LNCs) for topical delivery, which protected retinal morphology and function by reducing oxidative stress and apoptosis. These ultra-small nanocapsules (20 nm optimal size) sustained drug release and reduced oxidative stress and apoptosis, effectively protecting retinal morphology and function in NaIO3-induced damage models. The 20 nm formulation was optimal for retinal delivery, showing no irritation or toxicity in mouse and rabbit models.

Yadav et al. (2020) [25] formulated atorvastatin-loaded solid lipid nanoparticles (ATS-SLNs) as eye drops, significantly improving bioavailability, corneal flux, and stability over 12 months. The study included in vitro (ARPE-19, HCLE, R28, and RCE cells), ex vivo (for corneal permeability), and in vivo (in rabbits) evaluations, demonstrating prolonged drug release (96 h), superior ocular permeability, and safety in various ocular tissues, including the cornea, retina, and conjunctiva. ATS-SLNs were 8x more bioavailable in aqueous humor and 12x in vitreous humor than free ATS. Exploring the synergistic effects of gene therapy and antioxidant treatment for CNV in wet AMD, Li et al. (2023) [26] developed solid lipid nanoparticles (SLNs) modified with an NGR peptide for the co-delivery of microRNA-150 and quercetin. These nanoparticles (~200 nm) were stable in biological fluids and provided controlled drug release. They demonstrated dual mechanisms: angiogenesis inhibition via mRNA-150 and oxidative damage reduction by quercetin. The study, conducted in vitro on human umbilical vein endothelial cells (HUVECs) and in vivo in a mouse laser-induced CNV model, showed effective CNV growth inhibition for up to two weeks post-injection. The nanoparticles down-regulated angiogenesis markers (HIF-1α, CXCR4) and inflammation-related genes while enhancing cellular uptake of mRNA-150, while safety evaluations revealed no retinal toxicity, inflammation, or structural damage.

#### 2.1.2. Liposomes In Vivo

Liposomes are naturally occurring, amphiphilic bilayers of phospholipid molecules, whose structure is sealed, incessant, and vesicular, thus resembling biological membranes. They provide large surface areas and can enhance the solubility, controlled release, bioavailability, and precision targeting of the encapsulated material. Sun et al. (2024) [27] introduced dual-modified liposomes functionalized with penetratin and hyaluronic acid for topical delivery of conbercept, offering a non-invasive alternative for wet AMD treatment. These liposomes (~152 nm) enhanced intraocular drug delivery and achieved efficacy comparable to intravitreal injections in reducing CNV in laser-induced CNV mouse models. They exhibited excellent ocular biocompatibility, with no significant toxicity or irritation reported. This innovative design addresses the challenges of patient compliance by eliminating the need for invasive intravitreal injections. Davis et al. (2014) [28] formulated annexin A5 (AnxA5)-associated liposomes for the topical delivery of bevacizumab to the posterior segment of the eye in wet AMD. These liposomes, composed of phosphatidylcholine, phosphatidylserine, cholesterol, and α-tocopherol, were stable with no significant aggregation despite AnxA5 binding. In rat and rabbit models, AnxA5 enhanced corneal epithelial uptake and transcytosis, enabling efficient delivery of the drug to the posterior chamber. The liposomes showed no ocular surface modifications, conjunctival hyperemia, or other adverse effects, demonstrating the safety and feasibility of this non-invasive delivery system. RGD-modified liposomes (R: arginine; G: glycine; D: aspartic acid) were developed by Chen et al. (2024) [29], encapsulating 2-deoxy-D-glucose (2-DG) as a novel approach to treating wet AMD, particularly targeting endothelial cell metabolism to overcome resistance to anti-VEGF therapies. These liposomes (~133 nm, spherical) demonstrated enhanced targeting of neovascular endothelial cells. In laser-induced CNV mouse models, intravitreal injections led to significant reductions in CNV and vascular leakage by interfering with glycosylation. Additionally, endothelial cell proliferation, migration, and tube formation were effectively inhibited. Safety evaluations indicated no significant retinal or systemic toxicity, demonstrating the potential for safe and effective CNV treatment. Each approach was tailored to address specific challenges—targeting endothelial metabolism for CNV reduction [29], non-invasive drug delivery for patient compliance [27] and efficient posterior segment delivery via topical applications [28].

#### 2.1.3. Poly(Lactic-co-Glycolic Acid) (PLGA) NPs In Vivo

PLGA is one of the most widely used biodegradable polymers due to the low toxicity of its metabolite monomers, lactic acid and glycolic acid. There are many different forms of PLGA available with varying degradation times and monomer ratios. Different studies applied PLGA NPs, mostly for drug delivery, thus being the ones mostly reported in the literature used for AMD treatment applications. Suri et al. (2021) [30] designed PLGA NPs for subconjunctival injection. These nanoparticles (236.7–265.9 nm, biphasic release pattern) enhanced drug permeation through the sclera, improved cellular uptake, and demonstrated superior anti-angiogenic potential compared to uncoated nanoparticles. In vitro, ex vivo (goat scleral models), and in vivo (goat eyes) studies confirmed biocompatibility. Histopathological analysis revealed no irritation, while safety tests in HET-CAM and RBC models supported its clinical applicability for retinal degeneration diseases like AMD. Liu et al. (2019) [31] developed PLGA nanoparticles loaded with bevacizumab and dexamethasone using electrostatic conjugation. The nanoparticles (200 nm) provided controlled drug release and exhibited anti-angiogenic effects in in vitro studies using HUVECs and in vivo in chick embryo chorioallantoic membrane (CAM) and laser-induced rabbit CNV models. They effectively reduced VEGF secretion and CNV leakage, with no significant ocular toxicity reported, highlighting the combination therapy’s potential for CNV management. Self-assembled PLGA nanoparticles were developed by Xin et al. (2020) [32], which were encapsulated in calcium alginate hydrogels for topical treatment of wet AMD. These nanoparticles effectively penetrated ocular barriers and reduced CNV area by 51% in in vivo primate models (rhesus monkeys). The formulation was non-toxic and safer than steroid treatments. Qiu et al. (2019) [33] developed fenofibrate-loaded PLGA nanoparticles for the sustained treatment of AMD and diabetic retinopathy. The biodegradable nanoparticles demonstrated controlled drug release over 60 days, reduced VEGF and ICAM-1 expression, and ameliorated retinal dysfunction in in vivo (rats and Vldlr−/− mice) and in vitro models. Safety evaluations showed no toxicity or adverse effects, making them suitable for long-term therapy.

#### 2.1.4. Polymeric NPs In Vivo

Polymeric NPs, one of the most studied organic strategies for nanomedicine [34], allow for controlled drug release, protection of the agent against the environment, and improved bioavailability and therapeutic index. There are two types of polymeric NPs: nanocapsules, a reservoir system composed of an oily core and a polymeric shell, and nanospheres, a matrix system whose surface acts as a continuous network for drug retention or absorption [35]. Tsai et al. (2022) [36] evaluated hyaluronic-acid-coated gelatin nanoparticles (GEH + NPs) for non-invasive drug delivery to the posterior eye via topical and subconjunctival administration. These positively charged nanoparticles (253.4 nm, +9.2 mV) demonstrated high ocular bioavailability, with 6.89% accumulation in the posterior eye for topical use and 14.55% for subconjunctival injection. In vitro testing on ARPE-19 cells confirmed non-toxicity across 0.2–50 μg/mL concentrations, and in vivo studies on mouse models demonstrated efficient drug uptake with no cell death or adverse effects.

Polysaccharide nanoparticles (DSCS/XDSCS NPs) were investigated by Yang et al. (2021) [37] for the sustained delivery of neurotrophic factors (Oncostatin M and ciliary neurotrophic factor) to protect retinal ganglion cells and photoreceptors in models of retinal degeneration. These nanoparticles (~320–340 nm, negative surface charge, >91% encapsulation efficiency) provided neuroprotection for over 70 days following a single intravitreal injection. In vitro testing on photoreceptor progenitor cells and retinal ganglion progenitor cells derived from iPSCs and in vivo studies in rats of the Royal College of Surgeons (RCS, RP model) and a glaucoma model of rats (Optic nerve crush, ONC) confirmed significant neuroprotection without systemic toxicity. Aggregation at the injection site was noted but did not compromise efficacy. Liu et al. (2020) [38] designed cyclic RGD peptide-coated PLGA/PEI nanoparticles (cRGD-DPPNs) for co-delivery of dexamethasone and bevacizumab, targeting wet AMD with CNV. These nanoparticles (213.8 nm, PDI 0.153) demonstrated sequential drug release, reducing VEGF expression, CNV growth, and fluorescein leakage in a laser-induced rabbit CNV model. In vitro studies with ARPE-19 cells confirmed selective targeting of RPE cells via αVβ3 integrin overexpression, while HUVECs showed inhibition of migration, invasion, and angiogenesis. Histopathology confirmed no significant retinal toxicity.

Shen et al. (2024) [39] developed lutein/nintedanib co-assembled hybrid nanoparticles for minimally invasive treatment of CNV. These nanoparticles showed long-term sustained release for 1–2 months and significantly reduced angiogenesis, inflammation, and oxidative stress in in vivo laser-induced CNV mouse models and rabbit eyes. Subconjunctival injection proved as effective as intravitreal anti-VEGF therapy, with no significant adverse effects. This strategy offers improved patient compliance by reducing injection frequency. Yao et al. (2023) [40] created Angiopoietin1-anti-CD105-PLGA nanoparticles (AAP NPs) for targeted CNV therapy in wet AMD. These nanoparticles (213 nm, −33.81 mV zeta potential, 55.26% encapsulation efficiency) demonstrated controlled drug release for 28 days and enhanced accumulation at CNV sites in laser-induced rat CNV models. They reduced neovascularization leakage and inhibited VEGF and Ang2 secretion. Cytotoxicity tests confirmed >90% cell viability at concentrations below 10 mg/mL, with low apoptosis and necrosis rates.

Kim et al. (2010) [41] designed flexible biodegradable polymers, PLA/PLA-PEO (poly(ethylene oxide)/poly(l-lactic acid) nanoparticles encapsulating the C16Y peptide, an integrin antagonist, for sustained CNV inhibition. These nanoparticles (114–367 nm, −38.26 mV zeta potential) released the C16Y peptide for 6 weeks and reduced the CNV area more effectively than a peptide solution in rat laser-induced CNV models. The formulation showed no retinal toxicity or inflammation, with nanoparticles endocytosed by RPE cells within 24 h. Reis et al. (2022) [42] developed sodium butyrate-loaded chitosan-coated nanoparticles for intravitreal injection in CNV treatment. These nanoparticles (311 nm, +56.3 mV zeta potential, 92.3% encapsulation efficiency) exhibited sustained drug release over 35 days, inhibited angiogenesis in CAM assays, and showed no retinal toxicity or damage in in vivo rat models. In vitro studies on ARPE-19 cells confirmed biocompatibility and non-toxicity.

#### 2.1.5. Polysialic Acid-NPs (PolySia-NPs) In Vivo

Polysialic acid-decorated nanoparticles (PolySia-NPs) have emerged as a novel therapeutic platform. Engineered with a PEG-PLGA core and polysialic acid coating, these nanoparticles selectively bind receptors and modulate complement activity, targeting key inflammatory and immune pathways implicated in AMD progression. Krishnan et al. (2023) (2024) [43,44] conducted a series of studies to evaluate the therapeutic potential and safety of polysialic acid-decorated nanoparticles (PolySia-NPs) for AMD treatment, focusing on both geographic atrophy and exudative AMD with CNV. These nanoparticles (~100 nm) are composed of a PEG-PLGA core covalently attached to polysialic acid, selectively binding to human Siglec-7, -9, -11, and mouse Siglec-E receptors. Designed for targeted immune modulation, they reduce inflammation and preserve retinal structure by activating anti-inflammatory pathways. In vitro studies with human macrophages demonstrated that PolySia-NPs significantly reduced pro-inflammatory cytokines (TNF-α, IL-6, IL-1β, and VEGF) while increasing the anti-inflammatory cytokine IL-10. In a clinically validated, laser-induced CNV mouse model, PolySia-NPs reduced neovascular lesion size, reduced macrophage infiltration, and preserved retinal outer nuclear layer (ONL) thickness under high-intensity light exposure. Comprehensive in vitro and in vivo evaluations confirmed the safety of PolySia-NPs. In vitro studies indicated no mutagenicity or cytotoxicity. In vivo experiments using CD-1 mice and Sprague Dawley rats for intravenous administration revealed no clastogenic activity, systemic toxicity, or adverse effects on survival. Ocular toxicity studies in Dutch Belted rabbits and non-human primates demonstrated no significant toxicity after a single intravitreal (IVT) injection or two elevated repeat IVT doses administered seven days apart. Temporary, mild inflammation was observed in some cases but resolved without long-term effects. Histopathology showed no retinal structural changes or alterations in intraocular pressure. These findings validate the safety and biocompatibility of PolySia-NPs for intravenous and intravitreal administration, paving the way for the first-in-human clinical trials targeting geographic atrophy.

Peterson et al. (2024) [45] also investigated the use of PolySia-NPs for complement regulation as a therapy for wet AMD with CNV. These nanoparticles, functionalized with a polyethylene glycol linker, were designed to enhance complement factor H (CFH) binding to C3b, thereby inhibiting the alternative complement pathway. In vitro studies using human serum and M1 macrophages showed that PolySia-NPs reduced complement activity by decreasing hemolysis and C3b deposition and suppressed macrophage activation. In a laser-induced CNV mouse model, PolySia-NPs reduced microglial activation, complement-mediated inflammation, and neovascularization. The NPs were non-toxic to macrophages, microglia, and neural cells at therapeutic doses.

#### 2.1.6. Quantum Dots In Vivo

Quantum dots are nanoscale semiconductor particles with unique properties, making them highly versatile for biomedical applications. These nanoparticles, typically ranging from 2 to 10 nanometers in size, exhibit size-tunable fluorescence, high photostability, and excellent biocompatibility, enabling precise imaging, diagnostics, and therapeutic delivery, with potential to address complex pathologies like AMD. Huang et al. (2023) [46] developed an innovative MMP9-responsive graphene oxide quantum dot-based nano-in-micro drug delivery system (C18PGM) for combinatorial therapy targeting CNV in wet AMD. This system combines graphene oxide quantum dots (GOQDs), a highly biocompatible and antioxidative nanomaterial (~5 nm), with a peptide (C18P) and minocycline, designed for the targeted release of therapeutic agents at inflammatory sites where MMP9 is overexpressed. The C18P peptide ensures controlled drug release, addressing inflammation, oxidative stress, and VEGF-driven angiogenesis simultaneously. In a laser-induced CNV mouse model, an intravitreal injection of C18PGM significantly reduced CNV areas, and the combined therapy with bevacizumab exhibited superior antiangiogenic activity compared to either treatment alone. The system effectively suppressed inflammatory markers (TNF-α, IL-6, IL-1β) and reduced MMP9 expression, demonstrating modulation of the inflammatory microenvironment. Safety evaluations revealed no systemic toxicity, including no weight loss or liver and kidney dysfunction, and minimal retinal or systemic adverse effects, confirming a strong intraocular safety profile.

#### 2.1.7. Nanoceria In Vivo

Nanoceria, constituting cerium oxide-based nanoparticles, possess unique redox properties andbiocompatibility, and offer potential applications in oxidative stress management and drug delivery, which also applies to AMD. Badia et al. (2023) [47] demonstrated the therapeutic effects of cerium oxide nanoparticles (CeO₂NPs, 3 nm) administered topically via eye drops for both dry and wet AMD. In vitro studies using ARPE-19 cells showed reduced oxidative stress, while HUVEC studies confirmed inhibited neovascularization. In vivo, CeO₂NPs reached the retina in DKOrd8 mouse models, reducing VEGF levels, increasing PEDF levels, and reversing retinal transcriptome alterations. Additionally, laser-induced CNV lesions were significantly reduced, with decreased inflammation and retinal degeneration. The study supports the biocompatibility and efficacy of CeO₂NPs as an antioxidant, anti-inflammatory, and neuroprotective agent.

Nanoceria was also investigated by the group of Tisi et al. (2020 & 2022) [48,49] for dry and wet AMD, respectively. In the first study targeting dry AMD, Tisi et al. evaluated the effects of CeO₂NPs in a light-induced retinal damage model. In vitro, CeO₂NPs preserved ARPE-19 cell structure, reduced cell death, and downregulated oxidative stress and autophagy markers (LC3B-II, p62). In vivo intravitreal injections in rats reduced epithelial–mesenchymal transition and preserved RPE and photoreceptor cell structures. The study demonstrated CeO₂NPs’ biocompatibility, retention in the retina, and dual-action potential in targeting oxidative stress and autophagy pathways. For wet AMD, the researchers investigated nanoceria for mitigating retinal neovascularization. In vitro studies using ARPE-19 cells and HUVECs revealed reduced VEGF protein levels and oxidative stress-induced angiogenesis. In in vivo laser-induced CNV animal models, intravitreal injections of CeO₂NPs decreased choroidal neovascularization, suppressed vascular sprouting into the photoreceptor layer, and mitigated oxidative damage.

Mitra et al. (2017) [50] designed glycol chitosan-coated cerium oxide nanoparticles (GCCNPs) for wet AMD. These nanoparticles (~170 nm hydrodynamic size) suppressed VEGF expression and oxidative stress markers in ARPE-19 cells. In a mouse CNV model, intravitreal GCCNPs reduced CNV growth by ~40% and enhanced ROS scavenging. The nanoparticles demonstrated high aqueous solubility, autoregenerative antioxidant activity, and stability, with no cytotoxicity observed in in vitro or in vivo models. This dual-action formulation targeted oxidative stress and VEGF pathways effectively.

Wang et al. (2018, 2019) [51,52] developed and evaluated a nanoceria-loaded injectable hydrogel for treating AMD, focusing on its antioxidative and protective properties. In their 2018 study, the hydrogel demonstrated sustained nanoceria release over two months, effectively reducing ROS levels and inflammation while preventing retinal damage. In vitro experiments confirmed strong biocompatibility, reduced apoptosis, and enhanced antioxidative activity. The hydrogel matrix provided structural stability, protecting nanoceria from degradation and enabling autoregenerative cycling between Ce^3^⁺ and Ce^4^⁺ states for prolonged efficacy. In their subsequent 2019 study, the hydrogel significantly mitigated oxidative stress in a light-induced dry AMD mouse model, preserving retinal and photoreceptor structures and preventing further degeneration. Although some irreversible damage to the outer nuclear layer was observed, the hydrogel facilitated partial structural recovery, highlighting its potential as a long-term therapeutic strategy for AMD.

#### 2.1.8. Light-Sensitive NPs In Vivo

Recent developments in light-activated nanosystems for combination therapies targeting CNV in wet AMD show promise for NP use in AMD management. These advanced platforms integrate anti-angiogenic and photodynamic modalities. Light-responsive mechanisms, including red- or UV-light-triggered activation, enable ophthalmologists to achieve precise therapeutic effects with minimal systemic toxicity, representing a significant leap forward in AMD management. Xu et al. (2023) [53] developed a red-light-triggered photoactivatable nanosystem for combination anti-angiogenic and photodynamic therapy targeting CNV in wet AMD. This innovative system consists of a self-assembled nanoplatform combining an ROS-sensitive dasatinib (DAS) prodrug with the photosensitizer verteporfin (VER), coated with PEG-lipid for stability and efficient drug delivery. Under red light activation, the nanosystem releases DAS intraocularly while generating ROS, achieving simultaneous anti-angiogenic and photodynamic effects. In a laser-induced CNV mouse model, intravenous injection of the nanosystem significantly suppressed CNV progression, induced vascular occlusion, and reduced CNV leakage more effectively than monotherapies. In vitro experiments with HUVECs and ARPE-19 cells further confirmed its robust anti-angiogenic properties. Safety evaluations showed no significant systemic toxicity, as the prodrug remains inactive without light exposure, and no ocular damage was observed following irradiation. This light-activated approach ensures precise, localized therapy with minimal systemic side effects. Similarly, Huu et al. (2015) [54] designed a light-responsive polymeric nanoparticle depot for the controlled release of nintedanib, an angiogenesis inhibitor, in the posterior eye segment. These biocompatible, degradable nanoparticles are engineered to respond to far UV light, allowing for precise, ophthalmologist-controlled drug release. After intravenous injection, the nanoparticles stably retained their cargo in the vitreous and were triggered to release the encapsulated drug upon UV irradiation. In in vivo studies with CNV rat models, the system achieved CNV suppression with light-triggered drug release over an unprecedented duration of up to 30 weeks. This extended-release period far exceeds the typical 4–8-week interval between anti-angiogenic injections, highlighting its potential to reduce the frequency of treatments. Safety assessments, including electroretinograms (ERGs), corneal and retinal tomography, and histology, showed no adverse effects on ocular health.

Ideta et al. (2005) [55] formulated dendritic porphyrin encapsulated in polymeric micelles for photodynamic therapy (PDT) in CNV. These nanoparticles (~30 nm) exhibited targeted accumulation in CNV lesions with enhanced phototoxicity under irradiation compared to free porphyrin. In vivo studies in a rat laser-induced CNV model achieved up to 85% CNV closure rates with minimal off-target phototoxicity. The nanoparticles remained in retinal tissues for 3 days post-injection, ensuring localized efficacy. Krishnaswami et al. (2018) [56] aimed for a photosensitizer-loaded nanoparticle system and created PLGA nanoparticles loaded with hypocrellin B for photodynamic therapy. These nanoparticles (89.6–753.6 nm) generated significant ROS, leading to 85.5% cell death in A549 cells after irradiation. Anti-angiogenic effects were confirmed in CAM assays, and the in vivo models showed effective retina targeting with minimal systemic toxicity.

#### 2.1.9. Gene Therapy Delivery with NPs In Vivo

One promising strategy involves the use of intraceptor nanoparticles delivering recombinant plasmids intracellularly. These nanoparticles are designed for high specificity, stability, and extended-release properties, enabling sustained therapeutic effects with fewer interventions. Preclinical studies in murine and primate models have demonstrated significant regression of CNV, suppression of fibrosis, and vision restoration without ocular or systemic toxicity. Interceptor nanoparticles delivering Flt23k plasmids represent a cutting-edge gene therapy platform for AMD, with significant efficacy in reducing VEGF-mediated angiogenesis, CNV, and subretinal fibrosis. Both studies demonstrated long-lasting therapeutic effects, with Zhang et al. (2017) [57] emphasizing dose optimization and safety, while Luo et al. (2013) [58] highlighted extended-release properties and multi-species applicability, including primates. Safety profiles across studies confirmed no ocular or systemic toxicity, supporting the clinical translation of this technology. This approach provides a promising alternative to traditional anti-VEGF therapies, offering sustained, targeted treatment with fewer interventions. Zhang et al. (2017) [57] developed recombinant Flt23k intraceptor plasmid-loaded nanoparticles (RGD.Flt23k.NPs) for gene therapy targeting neovascular AMD. These nanoparticles aim to inhibit VEGF intracellularly, reducing CNV. Administered intravenously, the nanoparticles localized to CNV lesions and suppressed VEGF signaling with peak efficacy observed at 30–60 μg pFlt23k per mouse. In in vivo experiments using a laser-induced CNV mouse model, treatment significantly inhibited CNV volume and demonstrated robust anti-angiogenic effects. Safety evaluations showed no detectable toxicity or inflammation, no adverse effects on body weight, organ weights, visual function, hemoglobin levels, or complement C3 levels. The gene delivery system employed RGD-modified Flt23k plasmid-loaded nanoparticles, designed for high specificity and stability. This study highlights the potential of intraceptor nanoparticle therapy as a safer, long-lasting alternative to conventional anti-VEGF therapies for neovascular AMD. Luo et al. (2013) [58] investigated biodegradable nanoparticles delivering recombinant Flt23k intraceptor plasmid for extended-release gene therapy targeting wet AMD. These nanoparticles (~523–572 nm, zeta potentials −22.67 to −2.11 mV) achieved regression of CNV and suppression of subretinal fibrosis in both primate and murine models. Approximately 40% of vision was restored in murine models, demonstrating significant therapeutic efficacy. In in vivo studies involving laser-induced CNV models and non-human primates, the nanoparticles delivered sustained therapeutic effects and inhibited angiogenesis and fibrosis. Safety evaluations revealed no ocular or systemic toxicity, and no adverse health issues were observed in treated monkeys for at least 30 days post-treatment. Finally, Dasari et al. (2017) [59] proposed PEG-POD/DNA NPs (120–180 nm) for gene transfer in wet AMD and CNV, tested in mouse models through subretinal injections, leading to reduced choroidal neovascularization by 50% in the murine models, with efficient transgene expression in retinal cells in vivo and minimal immune response. Low cytotoxicity was proven in vitro (ARPE-19), and no significant adverse effects were observed in treated murine retinas. A reducible disulfide bond enabled controlled release of the non-viral NPs for targeted and controlled gene delivery.

#### 2.1.10. Dendrimers In Vivo

Dendrimers provide a platform for drug delivery in AMD, addressing challenges such as enzymatic degradation, off-target effects, and limited drug bioavailability. These highly branched, nanoscale polymers offer precise targeting capabilities and the ability to deliver therapeutics directly to tissues, reducing off-target effects while enhancing efficacy. Wu et al. (2023) [60] demonstrated the efficacy of dendrimers in stabilizing and systemically delivering peptide therapeutics, while Kambhampati et al. (2021) [61] showcased the ability of dendrimers to target inflammatory cells and RPE for improved anti-inflammatory and anti-angiogenic effects. Both studies underscore the safety of dendrimers, with minimal systemic or ocular toxicity. Wu et al. (2023) [60] developed a dendrimer-based nanoparticle system (D-ALG) for peptide delivery in wet AMD, addressing challenges such as enzymatic degradation and off-target binding while improving therapeutic efficacy. The system was designed to protect peptide payloads, maintaining 90% intact peptide after 1.5 h of in vitro exposure to enzymatic degradation. D-ALG also preserved antiangiogenic activity, as demonstrated by a reduction in endothelial vessel network formation in in vitro HUVEC cell studies. In in vivo rat models, systemic administration of D-ALG reduced CNV lesion area more effectively than ALG-1001 (a clinical-stage antiangiogenic peptide), underscoring its superior therapeutic potential. While no specific safety concerns were discussed, systemic administration achieved efficacy without reported adverse effects, highlighting the biocompatibility of the dendrimer system. Kambhampati et al. (2021) [61] investigated hydroxyl-terminated polyamidoamine dendrimers conjugated with triamcinolone acetonide (D-TA) for targeted suppression of inflammation and CNV in AMD. These dendrimers (5.4 ± 0.2 nm) were administered intravenously and demonstrated selective targeting of macrophages and RPE cells without requiring additional targeting ligands. In in vivo rat models, D-TA achieved significant suppression of CNV (>80%) and reduced inflammation, performing 50-fold better than the free drug. Safety evaluations showed minimal side effects, with no IOP elevation, cataract formation, or signs of systemic toxicity in vital organs. The system also showed potential in ex vivo human eye models, further validating its translational promise. This dendrimer-based approach highlights a potent and safe alternative for both early-stage (dry AMD) and late-stage (wet AMD with CNV) disease management.

#### 2.1.11. Nanoballs In Vivo

Nanoballs are a type of nanostructured delivery system specifically designed to enhance the stability, efficacy, and targeted delivery of therapeutic agents, such as small interfering RNA (siRNA), to specific tissues or cells. Ryoo et al. (2017) [62] developed siRNA-based anti-VEGF nanoballs (siVEGF nanoballs) for treating CNV in wet AMD, designed to deliver VEGF-targeting siRNA efficiently and safely while bypassing the TLR3 immune response to minimize inflammation. These nanoballs, approximately 259.82 nm in size with a zeta potential of −41.2 mV, consist of a siRNA hydrogel core condensed with polyethyleneimine (PEI) and coated with hyaluronic acid (HA) for enhanced stability and delivery. In the in vivo study using a laser-induced CNV mouse model and oxygen-induced retinopathy model, the nanoballs achieved significant VEGF inhibition, reducing CNV area by 26% compared to controls and silencing VEGF mRNA by 41% in the choroid and 61% in the retina, with effects lasting up to two weeks post-injection. The nanoballs showed no significant cytotoxicity in ARPE-19 cells and no retinal inflammation or structural damage in histological analysis up to 30 days post-injection.

#### 2.1.12. Nanoemulsions In Vivo

Nanoemulsions are advanced colloidal delivery systems comprising oil, water, and surfactants, where the droplet size typically ranges between 20 and 200 nanometers. These systems are thermodynamically or kinetically stable and are widely recognized for their ability to enhance the solubility, stability, and bioavailability of hydrophobic drugs. Huang et al. (2021) [63] evaluated fenofibrate-loaded nanoemulsion eye drops for treating CNV and macular edema. These eye drops increased drug concentration in ocular tissues, achieving higher drug levels in the vitreous and retina compared to systemic treatments. In in vivo models, including CNV in mice and diabetic retinopathy in rats, the nanoemulsions demonstrated significant efficacy in reducing retinal vascular leakage and inflammation. Safety evaluations revealed no structural or functional damage to the cornea or retina, confirming the nanoemulsion’s biocompatibility. Ge et al. (2020) [64] developed a penetratin-modified lutein nanoemulsion that forms an in situ gel upon eye drop administration for treating both wet and dry AMD. This innovative delivery system reduced retinal cell apoptosis from 31.98% to 2.05%, significantly decreased ROS levels in the retina, and improved retinal structure and electroretinography (ERG) readings in a sodium iodate-induced AMD mouse model. The encapsulation of lutein enhanced its solubility and stability, while the penetratin peptide modification improved ocular penetration and retinal localization. Safety assessments showed minimal ocular irritation and no significant morphological changes in ocular tissues after 7 days of administration.

#### 2.1.13. Nanofibers In Vivo

Nanofibers are ultra-thin fibers with diameters ranging from tens to hundreds of nanometers, produced through techniques like electrospinning. Their unique properties, including high surface-area-to-volume ratio, tunable porosity, and mechanical flexibility, make them versatile for a wide range of biomedical applications. Gao et al. (2023) [65] developed an injectable anti-inflammatory supramolecular nanofiber hydrogel containing betamethasone phosphate (BetP) for enhancing anti-VEGF therapy in wet AMD. The hydrogel forms via CaCl₂-triggered gelation and provides sustained drug release, effectively reducing choroidal neovascularization, inflammation, and oxidative stress in in vivo mouse CNV models. The system demonstrated self-healing properties and ROS-scavenging capabilities, further enhancing its therapeutic potential. Safety evaluations revealed minimal toxicity and no significant histopathological abnormalities in major organs, supporting the hydrogel’s biocompatibility.

#### 2.1.14. Gold NPs (AuNPs) In Vivo

Several studies have demonstrated the anti-angiogenic properties of gold nanoparticles (AuNPs), highlighting their potential as therapeutic tools for neovascular retinal diseases, like neovascular AMD. Roh et al. (2016) [66] investigated 20 nm neutral-charge AuNPs using both in vitro and in vivo models. In vitro, AuNPs inhibited VEGF-induced capillary network formation, cell proliferation, and phosphorylation of ERK1/2, Akt, and FAK in human umbilical vein endothelial cells (HUVECs). Importantly, no cytotoxicity or apoptosis was observed in ARPE-19 cells. In vivo, intravitreal injection of AuNPs significantly reduced the CNV area in laser-induced CNV mice, as confirmed by immunofluorescence staining showing reduced endothelial cell markers in treated lesions. Gold nanodiscs were explored by Song et al. (2017) [67] in oxygen-induced retinopathy models. In vitro studies demonstrated that GNDs inhibited migration and angiogenesis in human retinal microvascular endothelial cells (HRMECs). In vivo, GNDs reduced neovascularization, with higher concentrations yielding stronger effects. Safety assessments revealed no inflammation, apoptosis, or significant changes in ERG readings, indicating that GNDs are well tolerated. Shen et al. (2018) [68] explored the mechanism behind AuNP-induced anti-angiogenesis, identifying autophagy as a key factor. In vitro, AuNPs decreased HUVEC proliferation, migration, and capillary tube formation. In vivo, they reduced neovascularization in an oxygen-induced retinopathy model. Confocal microscopy and Western blotting demonstrated increased expression of autophagic markers in treated groups, suggesting that autophagy contributes to the therapeutic effects of AuNPs. Collectively, these studies highlight the diverse mechanisms by which gold nanoparticles exert anti-angiogenic effects, including VEGF pathway inhibition, autophagy induction, and laser-activated hyperthermic therapy. With their biocompatibility, safety, and selective accumulation in neovascular regions, gold nanoparticles show promise as innovative treatments for retinal vascular diseases. It should also be mentioned that gold nanoparticles are being widely used for imaging in AMD as contrast agents; however, the scope of this review focuses on therapeutic applications of NPs.

### 2.2. In Vitro Studies

#### 2.2.1. PLGA NPs In Vitro

PLGA NPs have a variety of applications in vitro, demonstrating potential for sustained drug delivery in AMD. Studies have shown their effectiveness in controlled release, VEGF inhibition, and biocompatibility, with advanced systems further reducing injection frequency and improving therapeutic outcomes. Narvekar et al. (2019) [69] developed axitinib-loaded PLGA nanoparticles to provide a sustained drug release system for wet AMD. The nanoparticles (131.33 ± 31.20 nm, zeta potential −4.63 mV, 87.9% entrapment efficiency) demonstrated a controlled drug release profile and effectively reduced VEGF expression in the in vitro study using ARPE-19 cells. The anti-angiogenic activity indicated the potential for long-term AMD therapy, while biocompatibility testing showed cytotoxicity below 12% at 10 μM concentrations. Bhatt et al. (2020) [70] developed resveratrol-loaded PLGA nanoparticles for intravitreal injection to treat AMD. These nanoparticles (102.7 nm, zeta potential −47.3 mV, 65.21% entrapment efficiency) showed sustained resveratrol release, effectively inhibiting VEGF expression in ARPE-19 cells.

Kelly et al. (2018) [71] formulated aflibercept-loaded PLGA nanoparticles (243.13 ± 17.64 nm, encapsulation efficiency 75.76%) for sustained drug delivery in AMD treatment. The nanoparticles released 75% of the drug over 7 days, compared to 24 h for aflibercept solution. Despite a slight reduction in ARPE-19 cell viability, no significant cytotoxicity was attributed to the encapsulated drug, making it a promising alternative to frequent intravitreal injections. The same group [72] also designed PLGA and chitosan nanoparticles for the delivery of doxorubicin to inhibit hypoxia-induced angiogenesis in AMD. In vitro studies in ARPE-19 cells demonstrated reduced HIF-1α and VEGF-A levels and lower cytotoxicity compared to free doxorubicin.

Rudeen et al. (2022) [73] developed a composite hydrogel combining PLGA microparticles and nanoparticles for dual delivery of aflibercept and dexamethasone. This injectable system provided extended drug release for 224 days in an in vitro study, maintaining bioactivity while reducing complications like endophthalmitis. Biodegradable hydrogel represents a significant advancement in reducing injection frequency for AMD patients. Hirani et al. (2016) [74] developed a thermoreversible gel containing triamcinolone acetonide-loaded PLGA nanoparticles (208 nm) for sustained VEGF inhibition in wet AMD. The formulation reduced VEGF secretion by 43.5% over 72 h, with no burst release, and sustained drug release for up to 10 days. Cytotoxicity testing showed no adverse effects, offering a safer alternative to free triamcinolone for reducing injection frequency. A thermoreversible gel with sunitinib PLGA NPs (~164.5 nm) was also created by Bhatt et al. (2019) [75] and assessed in vitro for neovascular AMD, showing high entrapment efficiency (72%), favorable zeta potential (−18.27 mV), enhanced anti-angiogenic potential, and VEGF inhibition compared to free sunitimib solution. The thermoreversible gel transitioned from liquid to gel at body temperature for extended retention in the vitreous humor. At therapeutic doses, low cytotoxicity and high cell viability (above 90%) were observed in ARPE-19 cells.

Magnetic nanoparticles (MNPs) have shown promise as both imaging agents and drug delivery systems. Their magnetic properties make them highly effective contrast agents for imaging, enabling precise visualization of retinal tissues. In AMD, MNPs functionalized with ligands or antibodies can target specific markers, such as vascular endothelial growth factor (VEGF), providing enhanced imaging for early diagnosis. Yan et al. (2018) [76] designed magnetic PLGA nanoparticles loaded with ranibizumab for AMD treatment. The nanoparticles (5–10 nm, spherical) effectively inhibited tube formation in HUVECs during in vitro angiogenesis assays, with minimal cytotoxicity. This system shows promise for enhanced targeted delivery using magnetic guidance, although further testing is needed to confirm its safety profile.

Elsaid et al. (2016) [77] introduced a “system-within-system” platform, embedding chitosan-based nanoparticles within PLGA microparticles for ranibizumab delivery. The nanoparticles (17–350 nm) demonstrated sustained drug release without burst, with high entrapment efficiency (69%). In in vitro studies, they retained ranibizumab bioactivity and structural integrity, showing no cytotoxicity in ARPE-19 and HUVECs.

#### 2.2.2. Biomimetic NPs In Vitro

McCormick et al. (2020) [78] combined PLGA or PGA nanoparticles with a synthetic Bruch’s membrane for dry AMD treatment. The nanoparticles (480 nm for PLGA, 59 nm for PGA) supported a healthy ARPE-19 cell monolayer without invasion or structural disruption. PLGA nanoparticles provided sustained drug release for 2 weeks, while PGA released drugs over 1 day. The biomimetic membrane was hydrophilic, exhibited high tensile strength, and mimicked the natural Bruch’s membrane. This platform offers dual functionality: delivering RPE cells and removing diseased retinal structures. Xia et al. (2022) [79] developed macrophage-disguised rapamycin-loaded nanoparticles (MRaNPs) for targeted treatment of CNV in AMD through intravenous administration. These biomimetic nanoparticles are coated with macrophage-derived cell membranes, leveraging macrophage-inherited properties to enhance targeting and therapeutic effects. The nanoparticles specifically accumulated in CNV lesions, downregulated the mTOR pathway, attenuated angiogenesis, and activated autophagy in RPE cells, highlighting their multifaceted therapeutic potential. In vitro studies with ARPE-19 and HUVECs confirmed the efficacy of MRaNPs, while in vivo tests in laser-induced CNV mouse models demonstrated significant reductions in angiogenesis and effective modulation of CNV pathology. Safety evaluations revealed no significant variations in liver and kidney function markers, normal nutritional markers, and no pathological changes in the retina or major organs.

#### 2.2.3. Polymeric NPs In Vitro

Khin et al. (2022) [80] developed Eudragit^®^ nanoparticles (EuNPs) loaded with Fenofibrate and stabilized with polyvinyl alcohol (PVA) and randomly methylated β-cyclodextrin (RMβCD) for topical ocular delivery, targeting AMD and diabetic retinopathy (DR). These nanoparticles (75–98 nm, positive zeta potential) exhibited improved Fenofibrate solubility and bioavailability, mucoadhesive properties, and sustained drug release following the Korsmeyer–Peppas model. In vitro studies using SIRC cell lines and artificial membranes confirmed cytocompatibility and moderate irritation levels and reduced hemolytic activity. The RMβCD complexation enhanced encapsulation efficiency, and the formulation showed significant potential for non-invasive AMD therapy. Liu et al. (2024) [81] explored the preparation of lutein-loaded nanoparticles synthesized via the Maillard reaction*, combining protein–saccharide conjugates (casein–saccharide) for retinal protection against sodium iodate (NaIO3)-induced damage. Saccharides such as mannose, galactose, lactose, maltose, dextran, and maltodextrin were used to enhance the nanoparticle properties. These spherical NPs demonstrated improved thermal stability and antioxidant capacity compared to free lutein. In in vitro studies using ARPE-19 cells, the nanoparticles effectively reduced cellular reactive oxygen species (ROS) production and stabilized mitochondrial membrane potential, preventing cell death under oxidative stress conditions. Oral administration was proposed, but further studies are needed to confirm in vivo efficacy. Wang et al. (2014) [82] investigated protein polymer NPs (elastin-like polypeptides, ELPs) engineered to deliver αB-crystallin mini-peptides as a therapeutic strategy for protecting human RPE cells against apoptosis and oxidative stress. These spherical nanoparticles (~30 nm) mimicked natural chaperone activity, stabilizing mitochondrial membrane potential and reducing apoptosis under oxidative stress conditions. In in vitro experiments, the nanoparticles demonstrated significant protective effects on RPE cells by preventing the nuclear translocation of αB-crystallin, a key stress response marker, and enhancing cell survival. The study highlighted the biocompatibility of ELP nanoparticles, showing no cytotoxic effects at therapeutic concentrations.

#### 2.2.4. Nanoemulsions In Vitro

Fernandes et al. (2022) [83] investigated the permeability, anti-inflammatory, and anti-VEGF effects of steroid-loaded cationic nanoemulsions in RPE cells under oxidative stress. These nanoemulsions, formulated with DABCO and quinuclidine-based surfactants, delivered triamcinolone acetonide (TA) effectively, improving solubility and intracellular trafficking. In in vitro models using ARPE-19 and ARPE-19/HMC3 monolayers, TA-loaded NEs reduced TNF-α and VEGF levels, indicating strong anti-inflammatory and anti-angiogenic activity. The NEs also demonstrated enhanced permeability in oxidative stress-induced RPE cells, suggesting potential as a non-invasive therapy for AMD. Characterized by a particle size of 218–242 nm, a zeta potential of 32–46 mV, and stability for four months, these cationic nanoemulsions were shown to be low in toxicity.

#### 2.2.5. Quantum Dots In Vitro

Qian et al. (2018) [84] developed a graphene quantum dot (GQD)-based drug carrier system encapsulated with β-cyclodextrin (β-CD) for the co-delivery of ranibizumab and bevacizumab, targeting macular degeneration, likely wet AMD due to its anti-angiogenic properties. These nano-carriers, less than 5 nm in size with uniform spherical and cubic-like structures, exhibited photoluminescence for potential bioimaging applications and demonstrated high stability, biocompatibility, and controlled drug release. The incorporation of β-cyclodextrin enhanced drug solubility and provided sustained drug release, with approximately 94% of the encapsulated drugs being released over time. In in vitro studies using the L929 mouse fibroblast cell line, the nano-carriers maintained high cell viability (>90%) over five days, confirming their biocompatibility and non-toxic nature.

#### 2.2.6. Gold NPs In Vitro

The studies on gold nanoparticles (AuNPs) highlight their significant potential as anti-angiogenic agents in the treatment of AMD. Hoshikawa et al. (2017) [85] focused on developing a novel delivery platform for a ranibizumab biosimilar using polyethylene glycol (PEG)-conjugated AuNPs with a core size of approximately 5 nm. These Mab/PEG-conjugated nanoparticles were successfully synthesized and optimized, achieving over 70% conjugation efficiency by varying PEG chain lengths (5 kDa and 10 kDa). Their in vitro experiments showed that these nanoparticles effectively inhibited tube formation of human endothelial cells in Matrigel assays. Interestingly, even PEG-conjugated AuNPs without the Mab inhibited tube formation, though no significant effect on cell proliferation was observed.

Wim H. De Jong and colleagues (2008) [86] explored the anti-angiogenic effects of gold nanoparticles by studying their impact on endothelial cell migration and tube formation. Using AuNPs with a diameter of 15 nm, they demonstrated that VEGF-induced cell migration in human umbilical vein endothelial cells (HUVECs) was significantly reduced in a dose-dependent manner, as evidenced by wound healing and Transwell assays. Furthermore, tube formation in Matrigel was strongly inhibited by the presence of AuNPs. Western blot analysis revealed a reduction in VEGF-induced phosphorylation of Akt and endothelial nitric oxide synthase (eNOS), signaling pathways critical for angiogenesis. Importantly, cytotoxicity tests confirmed that these nanoparticles did not affect cell viability.

Similarly, Chan et al. (2020) [87] investigated the inhibitory effects of small gold nanoparticles (3–5 nm) on VEGF-induced migration of choroid-retina endothelial cells (RF/6A). Using Transwell migration assays, they found that AuNPs dose-dependently prevented VEGF-induced cell migration, although no impact on cell adhesion to fibronectin was observed. Cytotoxicity assays verified that AuNPs did not reduce cell viability at tested concentrations. The study further demonstrated, through Western blotting, that AuNPs suppressed VEGF-induced phosphorylation of Akt and eNOS, supporting their anti-angiogenic properties. These findings indicate that AuNPs can inhibit cell migration via VEGF signaling without compromising cell adhesion or viability. Arvizo et al. (2011) [88] explored the role of AuNP size and surface charge in their anti-angiogenic activity. Using nanoparticles of 5, 10, and 20 nm sizes, they demonstrated that larger nanoparticles (20 nm) exhibited the strongest inhibition of VEGF165 and basic fibroblast growth factor (bFGF)-induced angiogenesis in HUVECs. Their results emphasized the importance of a naked nanoparticle surface in maximizing inhibitory effects, suggesting that size and surface properties are critical parameters for enhancing their therapeutic efficacy. Collectively, these studies underline the potential of gold nanoparticles as effective anti-angiogenic agents for AMD treatment.

#### 2.2.7. Niosomes In Vitro

Niosomes, non-ionic surfactant-based vesicles, are emerging as a versatile nanocarrier system in drug delivery and have been investigated recently on AMD by Dastaviz et al. (2024) [89], who explored the impact of umbelliprenin (UMB)-containing niosome nanoparticles on VEGF-A and CTGF gene expression in RPE cells as a potential therapeutic strategy for AMD. These niosomes demonstrated a significant reduction in VEGF-A and CTGF expression, highlighting their potential anti-angiogenic and anti-fibrotic properties. Compared to free UMB, UMB-niosomes achieved greater efficacy at lower dosages, with an IC50 of 25 µg/mL versus 96.2 µg/mL for free UMB. Their high entrapment efficiency and gradual drug release further enhanced therapeutic outcomes. Although the study focused on cellular effects, it underscored the advantages of UMB-niosomes over free UMB in reducing key gene expressions involved in AMD pathology.

#### 2.2.8. Silica NPs In Vitro

Silica NPs, having a tunable size, large surface area, and versatile surface chemistry, are also emerging in AMD treatment. Ultimo et al. (2023) [90] developed large-pore mesoporous silica nanoparticles (LP-MSNs) functionalized with polyethylenimine (PEI) for the non-invasive delivery of anti-VEGF siRNA in the treatment of CNV in wet AMD. These nanoparticles were engineered for efficient siRNA loading and controlled release, achieving a 70% reduction in VEGF expression in ARPE-19 cells. The functionalization with PEI facilitated endosomal escape and enhanced cellular uptake, while Rhodamine B (RhB) labeling allowed for nanoparticle tracking. The LP-MSNs, with a core size of ~105 nm and a hydrodynamic size of ~599 nm post-functionalization, featured a broad pore size (~17 nm) and a positively charged surface (+27.2 mV). This design provided high siRNA loading capacity and ensured sustained delivery. In vitro studies demonstrated minimal cytotoxicity, with only a ~20% reduction in cell viability at higher concentrations, and no significant hemotoxicity, indicating excellent biocompatibility.

#### 2.2.9. Liposomes In Vitro

Behroozi et al. (2018) [91] developed and tested in vitro “smart” liposomal NPs (<170 nm) for targeted drug delivery of oxidative stress in human embryonic stem-cell-derived RPE (hESC-RPE) cells, composed of diselenide-containing phospholipids, showing responsiveness to oxidative stress. The study was aimed at dry AMD oxidative stress management with redox-sensitive liposomes loaded with N-acetyl cysteine (NAC). The liposomes released NAC in the presence of oxidative stress, demonstrating increased cell survival and reduced ROS and no significant cytotoxicity.

### 2.3. In Silico—Radiosurgery Modeling Studies

Gold nanoparticles (AuNPs) hold significant promise for enhancing the efficacy and safety of stereotactic radiosurgery in AMD, particularly for neovascular AMD with CNV. These in silico preclinical studies highlight the dose-enhancing potential of AuNPs at the tissue interface, enabling targeted endothelial cell ablation with lower radiation doses with IRay™ System (Oraya Therapeutics, Inc.).

Brivio et al. (2015) [92] conducted a Monte Carlo simulation to evaluate the potential of gold nanoparticles (AuNPs) to enhance radiation dose delivery during kilovoltage stereotactic radiosurgery for neovascular AMD. Using 20 nm AuNPs at a concentration of 32 mg/g, the study achieved a dose enhancement ratio (DER) of 1.97 at the AuNP–tissue interface, significantly increasing the efficacy of endothelial cell ablation in CNV lesions. The AuNPs concentrated the radiation effect within 30 μm of their layer, minimizing the dose to surrounding tissues such as the retina, optic nerve, and neighboring organs, with a 49% reduction in off-target radiation exposure. Simulations showed consistent dose enhancement across various beam angular tilts and a stable effect at the irradiation site. These findings suggest that AuNP-enhanced radiotherapy could reduce required radiation doses while maintaining or improving efficacy for CNV treatment. The study supports integration with the IRay™ system, a clinically validated radiotherapy approach for AMD, which uses 100 kVp beams tilted at 30° to irradiate the macula. This approach could complement anti-VEGF therapies, potentially reducing treatment frequency.

Ngwa et al. (2012A) [93] explored the radiosensitization potential of AuNPs for stereotactic radiosurgery in neovascular AMD through dosimetric modeling. The study found a dose enhancement factor (DEF) ranging from 1.30 to 3.26 at AuNP concentrations of 1–7 mg/g for 100 kVp x-rays, with higher enhancement (up to 3.40) achieved at 80 kVp due to increased photoelectric interactions. The localized enhancement effect was confined to within 3 μm of AuNPs, effectively sparing healthy tissues such as the retina and optic nerve. Preclinical modeling showed that equivalent therapeutic doses could be achieved with reduced direct radiation exposure, suggesting significant dose-sparing potential. The study demonstrated size- and concentration-dependent efficacy. In another study, the same group, Ngwa et al. (2012B) [94], performed dosimetric modeling to refine the use of AuNPs as radiosensitizers in AMD treatment. This preclinical analysis focused on optimizing dose enhancement parameters for the IRay™ system’s 100 kVp beams, achieving a nucleus dose enhancement factor (nDEF) of 1.30–3.26 for AuNP concentrations of 1–7 mg/g. The enhancement effect was size- and concentration-dependent, with higher efficacy observed at lower x-ray energies. AuNPs enabled precise targeting of the cell nucleus (5 μm diameter), offering enhanced radiosensitization and confined effects to CNV lesions. The modeling supported the hypothesis that AuNPs could provide localized dose enhancement, reducing collateral damage to surrounding healthy tissues during stereotactic radiosurgery.

## 3. Discussion

AMD constitutes a significant challenge for ophthalmologists in terms of treatment due to the multifactorial nature of the disease and the available treatments. Nanomedicine offers a paradigm shift to the limited treatment options in practice, offering a safe and efficient therapeutic approach. This review highlights the spectrum of NPs applications for AMD treatment, which, however, also comes with challenges. A critical challenge lies in the limitations of preclinical models. Animal models, despite their utility, differ significantly from human eyes in anatomical and physiological features, leading to potential discrepancies in the biodistribution and overall behavior of NPs. These differences highlight the need for advanced in vivo studies that more accurately reflect human ocular conditions to better predict the safety and efficacy of NP-based therapies. All the previously mentioned studies are summarized below in Table 1.

Emerging technologies such as machine learning present promising opportunities to address the challenges of NP use in AMD. By analyzing large datasets, machine learning algorithms can identify subtle relationships between NP design parameters, biological interactions, and pharmacological outcomes. For example, these tools can model the effects of NP size, shape, and surface modifications on their ability to permeate the vitreoretinal interface, offering a predictive framework for optimizing NP design. Additionally, machine learning can accelerate high-throughput screening, enabling the rapid identification of optimal formulations tailored to individual patient needs, a step toward personalized medicine in AMD treatment.

Patents relating to the use of NPs for the treatment of retinal diseases indicate the intentions of leading pharmaceutical companies to deliver ocular drugs to the posterior segment and how these systems might be commercialized. NPs and nanogel compositions have been recently patented (US20230041815A1), describing the development of NPs and nanogel drug delivery systems utilizing biocompatible polymers such as PLGA and PEG to encapsulate therapeutic agents like sunitinib malate, a multi-receptor kinase inhibitor for AMD [95]. Another patent regarding NPs in AMD (US8168584B2) introduced methods for treating AMD using compstatin, a peptide that inhibits the complement system, and its analogs [96]. Kompella et al. (US 20090087494) patented compositions of nanocarrier systems made of poly(lactide-co-glycolide), poly(lactide), polycaprolactone, albumin, and chitosan for targeted delivery of anti-VEGF agents to the eye [97]. Venkatraman et al. (US20190133931A1) disclosed a patent for subconjunctival depot forming formulations for ocular drug delivery using liposomal formulations comprising one or more phospholipids in the manufacture of a drug for the treatment of posterior (e.g., AMD, macular edema, retinopathy) and/or anterior ocular segment diseases (e.g., glaucoma, cataract, or uveitis) [98].

Using active ingredients traditionally administered via intraocular injection, now formulated as nanoparticles, another patented intervention included “nanonized” active ingredients formulating disease-targeting nanoparticles—such as liposomes, micelles, and lipid nanospheres—coated with VEGF-targeting agents for precise anti-inflammatory and anti-angiogenic action (WO2018162271A1 [99], US20200262903A1 [100]). Other notable innovations involve the use of photoactive nanoparticles for restoring vision (US20200113843A1) [101], the delivery of IL-33 via adeno-associated virus (AAV) vector gene therapy for managing AMD (WO2022074370A1) [102], and pharmaceutical formulations with inorganic nanoparticles selected from titanium oxide or silica nanoparticles as an active ingredient for angiogenesis-related conditions, including age-related macular degeneration (US20140044753A1) [103]. Sustained delivery systems using corticosteroid-encapsulated nanoparticles incorporated within thermoreversible hydrogels (US10729663B1) [104] and griseofulvin-based microparticles and nanoparticles (US2023248689A1) [105] are designed to reduce the frequency of injections while ensuring prolonged therapeutic efficacy. Furthermore, multifunctional nanoparticles, comprising a nanomaterial tethered to two ligands, are designed for use not only as treatment but also as a diagnostic agent for a range of diseases, including AMD (US2022409743A1) [106]. AMD treatment has also been advanced by the use of cell-penetrating peptides (CPPs) to deliver therapeutic agents such as anti-dyslipidemic, anti-inflammatory, and anti-angiogenic compounds through CPP-conjugated nanoparticles, micelles, or liposomes (US10905770B2) [107]. This non-invasive approach, administered as eye drops or contact lenses, reduces side effects and eliminates the need for intraocular injections. Another notable innovation includes the use of anthracycline antibiotics in targeted, sustained-release drug delivery systems such as nanoparticles, microspheres, and hydrogels to inhibit HIF-1 activity, effectively treating AMD symptoms with effects lasting up to two months per administration (CN103083341A) [108].

Finally, NP-based treatments hold great promise for the future of AMD treatment with multiple investigations of different NPs in various administration ways and targeting diverse pathways or disrupted mechanisms related to AMD. Oxidative stress, inflammation, angiogenesis, biomimicry, and mostly drug delivery are the focus of NP use for AMD. The enhancement of radiotherapy with light-sensitive NPs is also a promising and innovative approach of NP use, which can benefit AMD patients. Wet AMD is in the spotlight of investigations, but there are research efforts for dry AMD as well, as shown in our review. Longer-lasting drug bioavailability, sustained release, and minimal adverse effects are demonstrated across studies, in comparison to the high frequency of intravitreal injections. However, clinical trials in humans are yet to be conducted and show real-world results of NP-based therapies.

## 4. Limitations

While nanomedicine offers promising advancements in the treatment of AMD, several limitations and challenges must be addressed before clinical implementation. One of the primary concerns is the potential toxicity and long-term safety of NPs in human clinical trials. The risks of bioaccumulation, inflammatory response, influence of ocular anatomy [109], and unintended local and off-target effects remain unclear.

Several studies have attributed ocular toxicity to NPs, potentially via oxidative stress, mitochondrial damage, and apoptosis, although this is the very mechanism that most researchers aim to tackle in AMD therapy, as shown in this review. NPs can affect key structures such as the cornea, retina, optic nerve, and macula [110]. Organic NPs, such as polymeric and liposomal NPs, generally exhibit low toxicity, although surface modifications influence their safety [111]. Quantum dots have demonstrated corneal retention and potential long-term toxicity [112]. Inorganic NPs, including gold, silver, copper, cerium oxide, titanium dioxide, and zinc oxide NPs, can induce retinal damage, neurotoxicity, and genotoxic effects, often mediated by excessive reactive oxygen species (ROS) production and inflammatory responses [113]. While some NPs, such as PEGylated PLGA and liposomes show minimal toxicity, others like titanium dioxide NPs, cause significant cytotoxicity and corneal damage and impair the inner blood–retinal barrier [114,115]. Long-term studies are essential to assess chronic exposure risks, particularly in patients requiring sustained NP-based drug delivery. Moreover, the route that NPs follow after administration remains uncertain, as they may accumulate in retinal cells or migrate beyond the ocular environment, leading to systemic exposure and unknown long-term effects.

Another major challenge is the scalability and manufacturability of NP-based therapies. From laboratory-scale production to large-scale manufacturing, high-quality control measures for reproducibility are needed. High precision is required to maintain consistency in NP size, composition, and surface modifications, which are critical for ensuring reproducibility and efficacy. Additionally, cost efficiency remains a barrier and raises concerns about affordability and accessibility. Many NP formulations involve expensive synthesis techniques and specialized equipment. Simultaneously, the regulatory landscape presents obstacles, particularly regarding classification, safety evaluation, and approval processes with regulatory bodies such as the U.S. Food and Drug Administration (FDA) and the European Medicines Agency (EMA) imposing strict guidelines for NP-based therapeutics, added to the complexity of Good Manufacturing Practices (GMP) compliance.

Patient-related factors also complicate the clinical application of nanomedicine in AMD treatment. Interpatient variability, including differences in age, genetic predisposition, disease stage, and systemic comorbidities, may influence the pharmacokinetics and therapeutic response of NPs. For instance, variations in retinal permeability and immune responses could lead to inconsistent treatment outcomes [116]. Advanced approaches such as targeted drug delivery systems and personalized nanomedicine may mitigate some of these issues, paving the way for safer and more effective AMD therapies.

## 5. Conclusions

The integration of nanomedicine in AMD management not only provides innovative solutions to overcome current therapeutic limitations but also shows potential in enhancing outcomes and patient quality of life. Nanoparticle-based therapeutic options can be transformative for AMD management, causing a paradigm shift for the benefit of AMD patients. NPs are pre-clinically proving an effective, targeted, and precise therapeutic option. However, a deeper understanding of NP–biological interactions is essential to translate these technologies from bench to bedside. Therefore, clinical trials and the synergistic use of technological innovations are the next promising step in the long but worthwhile way to changing AMD management.

## Figures and Tables

**Table 1:** Summarized studies investigating nanoparticles in AMD treatment.

Study	NP Category	Use of NPs	AMD Type	Proposed Administration	Study Design
[21]	Lipid NPs	Drug delivery (dexamethasone)	Dry and wet AMD	Intravitreal injection	in vivo
[22]	Lipid NPs	Drug co-delivery (artemisinin and dexamethasone)	Wet AMD	Topical (eye drops)	in vitro and in vivo
[23]	Lipid NPs	Drug delivery (rapamycin)	Not specified	Intravitreal injection	in vitro and in vivo
[24]	Lipid NPs	Drug delivery (astragaloside IV)	Dry AMD	Topical (eye drops)	in vivo
[25]	Lipid NPs	Drug delivery (atorvastatin)	Not specified	Topical (eye drops)	in vitro, ex vivo, and in vivo
[26]	Lipid NPs	Drug co-delivery (mRNA-150 and quercetin)	Wet AMD, CNV	Intravitreal injection	in vitro and in vivo
[29]	Liposomes	Drug delivery (2-deoxy-D-glucose (2-DG)	Wet AMD	Intravitreal injection	in vivo
[27]	Liposomes	Drug delivery (conbercept)	Wet AMD	Topical (eye drops)	in vivo
[91]	Liposomes	Drug delivery (N-acetyl cysteine)	Dry AMD	Topical or intravitreal injection	in vitro
[28]	Liposomes	Drug delivery (bevacizumab)	Wet AMD	Topical (eye drops)	in vivo
[68]	PLGA NPs	Drug delivery (axitinib)	Wet AMD	Intravitreal injection	in vitro
[30]	PLGA NPs	Drug delivery (sirolimus)	Wet AMD	Subconjunctival injection	in vivo, ex vivo and in vitro
[69]	PLGA NPs	Drug delivery (resveratrol)	Wet AMD	Intravitreal injection	in vitro
[75]	PLGA NPs	Biomimetic (Bruchs membrane)	Dry AMD	Not specified	in vitro
[79]	PLGA NPs	Biomimetic and drug delivery (rapamycin)	CNV	Intravenous injection	in vitro and in vivo
[31]	PLGA NPs	Drug delivery (bevacizumab)	CNV	Intravitreal injection	in vitro and in vivo
[70]	PLGA NPs	Drug delivery (aflibercept)	Not specified	Not specified	in vitro
[74]	PLGA NPs	Drug delivery (rabibizumab)	Wet AMD	Intravitreal injection	in vitro
[33]	PLGA NPs	Drug delivery (fenofibrate)	CNV	Intravitreal injection	in vivo and in vitro
[56]	PLGA NPs	Photodynamic therapy dose-enhancement	Not specified	Intravenous injection	in vitro and in vivo
[71]	PLGA NPs	Drug delivery (anti-VEGF and corticosteroid)	Wet AMD, CNV	Injectable hydrogel	in vitro
[32]	PLGA NPs	Immune modulation	Wet AMD, CNV	Topical (eye drops)	in vivo
[75]	PLGA NPs	Drug delivery (sunitinib)	Wet AMD	Thermoreversible gel	in vitro
[72]	PLGA NPs	Drug delivery (doxorubicin)	Wet AMD	Intravitreal injection	in vitro
[72]	PLGA NPs	Drug delivery (triamcinolone acetonide)	Wet AMD, CNV	Thermoreversible gel	in vitro
[73]	PLGA NPs	Drug delivery (ranibizumab)	Wet AMD	Not specified	In vitro
[77]	Polymeric NPs	Drug delivery (fenofibrate)	Not specified	Topical (eye drops)	in vitro
[36]	Polymeric NPs	Drug delivery (gelatin-epigallocatechin gallate NPs)	Not specified	Topical or subconjunctival	in vivo and in vitro
[37]	Polymeric NPs	Drug delivery (oncostatin M and CNF)	Not specified	Intravitreal injection	in vitro and in vivo
[38]	Polymeric NPs	Drug co-delivery (dexamethasone and bevacizumab)	Wet AMD, CNV	Intravitreal injection	in vivo
[78]	Polymeric NPs	Drug delivery (lutein)	Not specified	Oral administration	in vitro
[82]	Polymeric NPs	Anti-inflammatory agent (elastin-like-polypeptides)	Not specified	Not specified	in vitro
[39]	Polymeric NPs	Drug co-delivery (lutein and nintedanib)	CNV	Subconjunctival injection	in vivo
[40]	Polymeric NPs	Biomimetic (angiopoietin1-anti CD105 NPs)	CNV	Intravenous injection	in vitro and in vivo
[42]	Polymeric NPs	Drug delivery(sodium butyrate)	Wet AMD, CNV	Intravitreal injection	in vitro and in vivo
[41]	Polymeric NPs	Drug delivery (integrin-antagonist peptide)	CNV	Intravitreal injection	in vitro and in vivo
[55]	Polymeric NPs	Photodynamic therapy dose-enhancement	Wet AMD, CNV	Intravenous injection	in vivo
[59]	Polymeric NPs	Drug delivery (gene therapy—hFLT1 gene)	Wet AMD, CNV	Subretinal	in vitro and in vivo
[44]	PolySia-NPs	Immune modulation	Geographic atrophy	Intravenous/intravitreal injection	in vitro and in vivo
[43]	PolySia-NPs	Immune modulation	CNV	Intravitreal injection	in vitro and in vivo
[45]	PolySia-NPs	Immune modulation	Wet AMD, CNV	Intravitreal injection	in vitro and in vivo
[46]	Quantum Dots	Antioxidant (Graphene Oxide QDs), drug delivery (minocycline)	Wet AMD	Intravitreal injection	in vivo
[81]	Quantum Dots	Drug co-delivery (ranibizumab and bevacizumab)	Wet AMD	Intravitreal injection	in vitro
[47]	Nanoceria	Antioxidant (CeO₂)	Dry and wet AMD	Topical (eye drops)	in vitro and in vivo
[49]	Nanoceria	Antioxidant (CeO₂)	CNV	Intravitreal injection	in vitro and in vivo
[48]	Nanoceria	Antioxidant (CeO₂)	Dry AMD	Intravitreal injection	in vitro and in vivo
[50]	Nanoceria	Antioxidant (Glycol chitosan-coated CeO₂ NPs)	Wet AMD	Intravitreal injection	in vitro and in vivo
[52]	Nanoceria	Drug delivery	Dry AMD	Injectable hydrogel	in vivo
[51]	Nanoceria	Antioxidant (CeO₂)	Dry and wet AMD	Injectable hydrogel	in vitro
[53]	Light-sensitive NPs	Drug delivery (anti-angiogenic and photosensitizer)	CNV	Intravenous injection	in vitro and in vivo
[54]	Light-sensitive NPs	Drug delivery (nintedanib)	CNV	Intravenous injection	in vitro and in vivo
[57]	Gene therapy NPs	Drug delivery (gene therapy- Flt23k)	Wet AMD	Intravenous injection	in vivo
[58]	Gene therapy NPs	Drug delivery (gene therapy—Flt23k)	Wet AMD	Intravenous injection	in vivo
[60]	Dendrimers	Drug delivery (peptide-based therapeutics)	Wet AMD, CNV	Intravitreal injection and systemic	in vitro and in vivo
[61]	Dendrimers	Drug delivery (triamcinolone)	Dry and wet AMD	Intravenous injection	in vivo and ex vivo
[61]	Nanoballs	Drug delivery (anti-VEGF siRNA)	Wet AMD, CNV	Intravitreal injection	in vivo
[80]	Nanoemulsions	Drug delivery (triamcinolone Acetonide)	Not specified	Not specified	in vitro
[62]	Nanoemulsions	Drug delivery (fenofibrate)	CNV	Topical (eye drops)	in vivo
[63]	Nanoemulsions	Drug delivery (lutein)	Dry and wet AMD	Eye drops/in situ gel	in vivo and in vitro
[64]	Nanofibers	Drug delivery (betamethasone)	Wet AMD, CNV	Injectable hydrogel	in vivo
[88]	AuNPs	Radiation dose-enhancement	Wet AMD, CNV	Not specified	in silico
[90]	AuNPs	Radiation dose-enhancement	CNV	Intravenously or localized targeting	radiosurgery modeling
[89]	AuNPs	Radiation dose-enhancement	CNV	Localized targeting	radiosurgery modeling
[65]	AuNPs	Anti-angiogenic therapy	Not specified	Intravitreal injection	in vitro and in vivo
[66]	AuNPs	Anti-angiogenic therapy	Not specified	Not specified	in vitro and in vivo
[67]	AuNPs	Anti-angiogenic therapy	Not specified	Not specified	in vitro and in vivo
[86]	Niosomes	Drug delivery (umbelliprenin)	Not specified	Not specified	in vitro
[87]	Silica NPs	Drug delivery (anti-VEGF siRNA)	Wet AMD, CNV	Topical (eye drops)	in vitro

NPs: Nanoparticles; AMD: Age-related Macular Degeneration.

## Data Availability

Not applicable.
